# Therapeutic Interventions for Apocrine, Eccrine, and Pseudo Chromhidrosis: An Overview of the Current Literature

**DOI:** 10.1111/srt.70167

**Published:** 2025-05-05

**Authors:** Jimmy Dhillon, Madeline Tchack, Bassem Rafiq, Noah Musolff, Babar Rao

**Affiliations:** ^1^ Rutgers Robert Wood Johnson Medical School New Brunswick New Jersey USA; ^2^ Rao Dermatology Atlantic Highlands New Jersey USA; ^3^ Department of Dermatology Rutgers Robert Wood Johnson Medical School New Brunswick New Jersey USA; ^4^ Department of Dermatology Weill Cornell Medicine New York New York USA

1

To the Editor,

Chromhidrosis (CH) is a rare condition characterized by the secretion of colored sweat. Recently, the characteristics and treatment outcomes of CH were summarized [1]. However, there remains a gap in the literature reviewing the current therapeutic interventions for CH. Here we present an overview of the treatment options available for the three subtypes of CH: apocrine chromhidrosis (ACH), eccrine chromhidrosis (ECH), and pseudochromhidrosis (PCH) (Table 1). Reports of CH that were successfully treated were found in PubMed and Scopus using the search terms “chromhidrosis AND treatment” and “colored sweat AND treatment.”

**TABLE 1:** The mechanisms of action of and duration of symptom relief provided by the treatment options for apocrine chromhidrosis, eccrine chromhidrosis, and pseudochromhidrosis.

	Treatment	Mechanism of action	Duration of symptom relief
Apocrine chromhidrosis	Mechanical pressure	Releases contents of apocrine glands	Up to three days
	Topical capsaicin	Reduces and prevents reaccumulation of substance P	Symptoms return after treatment discontinuation
	Antiperspirants (20% aluminum chloride hexahydrate solution)	Prevents perspiration	Symptoms return after treatment discontinuation
	Botulinum Toxin Type‐A	Prevents acetylcholine release at the pre‐synaptic motor neuromuscular plate and inhibits substance P release	Up to 19 weeks post‐treatment
Eccrine chromhidrosis	Address causative agent (food dyes, drugs containing colored dyes such as tartrazine, hyperbilirubinemia)	Removes the source of the water‐soluble compounds released in eccrine glands	Resolution of symptoms
Pseudochromhidrosis	Address causative agent (chromogenic bacteria, dyes, chemical agents, and drugs)	Removes the source causing the pigmentation of colorless sweat	Resolution of symptoms

ACH involves the secretion of colored sweat from apocrine glands, most commonly in the axilla [2]. Applying manual pressure to release the contents of the gland can relieve symptoms for up to three days [3]. Topical capsaicin, when applied once to twice daily, can also result in the suppression of ACH [2]. Capsaicin acts by reducing and preventing the reaccumulation of substance P, a chemomediator of pain in unmyelinated, slow‐conducting type C sensory fibers of the peripheral nervous system. The role of substance P in sweat production has been hypothesized to be due to its vasodilatory properties and since substance P‐positive nerve fibers have been found near sweat glands. However, symptoms can return after the discontinuation of therapy. Antiperspirants, such as a 20% aluminum chloride hexahydrate solution, can provide temporary relief as well [2, 3, 4]. Lastly, botulinum toxin type‐A (BTX‐A) has been shown to be effective in the treatment of facial apocrine chromhidrosis for up to 19 weeks post‐treatment [4]. BTX‐A prevents acetylcholine release at the pre‐synaptic motor neuromuscular plate, blocking parasympathetic stimulation necessary for sweat glands. Similar to topical capsaicin, BTX‐A has also been shown to inhibit substance P release.

ECH occurs when water‐soluble compounds are secreted through eccrine glands [4]. Thus, it is important to address the underlying cause for treatment. Examples of possible etiologies of ECH include the ingestion of food dyes, ingestion of drugs containing colored dyes (such as tartrazine), and hyperbilirubinemia [3].

PCH is the result of colorless sweat becoming pigmented at the skin surface due to chromogenic bacteria, dyes, chemical agents, and drugs [3, 5]. Similar to ECH, it is important to remove the causative agent to treat PCH. Most cases of PCH due to chromogenic bacteria can be treated with a combination of topical and oral erythromycin, although PCH due to *Serratia marcescens* might require a combination of topical nadifloxacin and oral cefcapene pivoxil [3, 5].

Overall, to aid clinical practice, we summarize the current treatment options for CH, as well as their mechanisms of action and duration of symptom relief. Dermatologists should be aware of the etiologies underlying the different subtypes of CH, as the treatments vary between subtypes, as well as in their efficacy. Furthermore, dermatologists should address the psychosocial impact of CH. Since patients can experience social and emotional distress, it is important to reassure patients that their symptoms do not necessarily signify an underlying systemic process, especially in the cases of ECH and PCH.

## Conflicts of Interest

The authors declare no conflicts of interest.

## Data Availability

The authors have nothing to report.
